# Exploring country-wide equitable government health care facility access in Uganda

**DOI:** 10.1186/s12939-020-01371-5

**Published:** 2021-01-18

**Authors:** Nicholas Dowhaniuk

**Affiliations:** 1 Department of Geography, University of Florida, 3141 Turlington Hall, 330 Newell Dr, Gainesville, FL 32601 USA; 2 Department of Environmental and Global Health, University of Florida, Gainesville, USA; 3 Tropical Conservation and Development Program, University of Florida, Gainesville, USA

**Keywords:** Accessibility analysis, AccessMod 5.0, Bicycles, Equity, Health care, Social determinants of health, Sub-Saharan Africa, Uganda

## Abstract

**Background:**

Rural access to health care remains a challenge in Sub-Saharan Africa due to urban bias, social determinants of health, and transportation-related barriers. Health systems in Sub-Saharan Africa often lack equity, leaving disproportionately less health center access for the poorest residents with the highest health care needs. Lack of health care equity in Sub-Saharan Africa has become of increasing concern as countries enter a period of simultaneous high infectious and non-communicable disease burdens, the second of which requires a robust primary care network due to a long continuum of care. Bicycle ownership has been proposed and promoted as one tool to reduce travel-related barriers to health-services among the poor.

**Methods:**

An accessibility analysis was conducted to identify the proportion of Ugandans within one-hour travel time to government health centers using walking, bicycling, and driving scenarios. Statistically significant clusters of high and low travel time to health centers were calculated using spatial statistics. Random Forest analysis was used to explore the relationship between poverty, population density, health center access in minutes, and time saved in travel to health centers using a bicycle instead of walking. Linear Mixed-Effects Models were then used to validate the performance of the random forest models.

**Results:**

The percentage of Ugandans within a one-hour walking distance of the nearest health center II is 71.73%, increasing to 90.57% through bicycles. Bicycles increased one-hour access to the nearest health center III from 53.05 to 80.57%, increasing access to the tiered integrated national laboratory system by 27.52 percentage points. Significant clusters of low health center access were associated with areas of high poverty and urbanicity. A strong direct relationship between travel time to health center and poverty exists at all health center levels. Strong disparities between urban and rural populations exist, with rural poor residents facing disproportionately long travel time to health center compared to wealthier urban residents.

**Conclusions:**

The results of this study highlight how the most vulnerable Ugandans, who are the least likely to afford transportation, experience the highest prohibitive travel distances to health centers. Bicycles appear to be a “pro-poor” tool to increase health access equity.

**Supplementary Information:**

The online version contains supplementary material available at 10.1186/s12939-020-01371-5.

## Background

Substantial health care disparities exist between much of rural and urban Sub-Saharan Africa (SSA). The majority of health care resources are located within urban centers, even in predominantly rural countries [1, 2]. Rural communities have lower health care and clinical laboratory access, higher levels of poverty and unemployment, and longer travel times to social service providers [1–4]. Lack of health care equity in SSA has become of increasing concern as many countries have entered a period of simultaneous high infectious and non-communicable disease burdens [5–7], the second of which requires a robust primary care network to diagnose, treat, and monitor patients due to a long continuum of care [8].

Health care inequities are addressed through supply-side and demand-side factors. Supply-side factors are characteristics directly related to the health system itself, including drug and equipment shortages, the physical location of health centers (HCs), and lack of qualified human resources. Demand-side factors are characteristics directly associated with the end-user, including barriers related to distance and travel time to a health facility, lack of transportation to obtain care, and lack of confidence in the quality of services [9]. Demand-side barriers have a negative impact on health care utilization among the poor, including linkages to the continuum of HIV care [10], family planning services [11], and maternal health care services [12].

Health systems in Uganda have been criticized for being inequitable [13], with the poor receiving less services than needed, and the rich receiving more than needed [14]. Qualitative evidence has shown distance to government health facilities is a barrier both due to a facility being too far to travel to, as well as the economic opportunity costs associated with travel to the health facility [15]. Similarly, restricted geographic access to health facilities influences poorer Ugandans to seek care at the nearest health facility or provider, even if the quality of care is lower [16]. The interplay of poverty and health care access has been cited in numerous studies on health care utilization among Ugandans [17], leading to increased reliance on traditional, family, and community sources of health care instead of seeking professional care [18, 19]. However, the relationship between poverty and health care access has not been modelled quantitatively on a country-wide basis.

Transportation-related barriers pose significant challenges to health care among the poor in Uganda [20]. Bicycle ownership has been proposed and promoted by Non-Governmental Organizations (NGOs) and Social Enterprises as one tool to reduce travel-related barriers to health-services (among other things, such as market access) through the distribution of cheap and durable bicycles and bicycle ambulances. While bicycle ownership has been associated with increased utilization of maternal health services [21], no analyses exist (to the author’s knowledge) that quantify and examine the relationship among poverty and the potential time savings bicycles offer compared to walking scenarios in accessing health care services on a country-wide basis. Understanding where low levels of accessibility to health care are prevalent, as well as quantifying the relationship between poverty and health care access, is vital for intentional resource allocation for bicycle programs towards the most vulnerable individuals to address unequitable health care systems [22, 23]. Thus, in this paper, the impact on government health facility access in Uganda is investigated through a health facility accessibility analysis, spatial statistics, random forests modeling, and linear mixed-effects models to answer the following questions: 1) is access to government health care facilities in Uganda equitable?, 2) to what proportion of the Ugandan population do bicycles increase one-hour access to government health facilities in Uganda?, 3) is access to government health services equitable for rural and poor residents of Uganda? and 4) does increased health unit access through bicycle ownership benefit the poor?

## Methods

### Study area

**Fig. 1:**
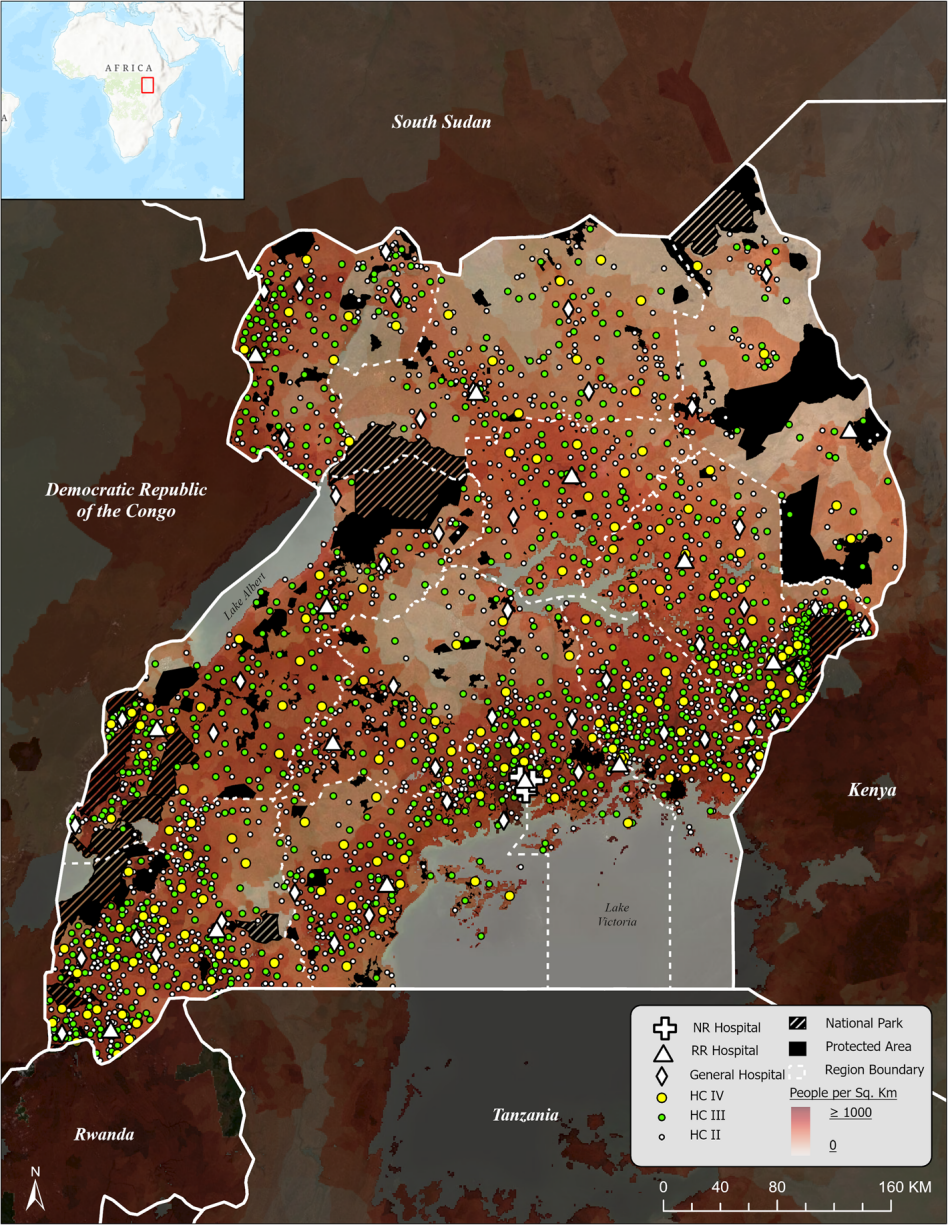
Study area map of Uganda with National Referral (NR) Hospital, Regional Referral (RR) Hospital, General Hospital, and Health Center (HC) locations in relation to Population Density

Uganda is located in East Africa with a population of approximately 41 million people in a country that is approximately 241 km^2^ in area. Kampala, the capital city, with a population of approximately 1.35 million, has the majority of higher level health services, even though 84% of Ugandans live in rural areas [24]. The health system in Uganda operates on a decentralized referral system [25]. A patient’s first point of contact within the health system is often through the Village Health Team (VHT), who are responsible for basic health interventions within local communities and villages. A VHT will refer a patient to a higher-level HC facility depending on the required complexity of services (Table 1). Patients are generally referred to the nearest HC that has the capacity and resources to treat or diagnose their illness. Higher level facilities tend to be in urban centers, with the National Referral Hospitals (NRHs) located in Kampala, while lower level health facilities are more widely distributed among rural communities (Fig. 1). According to the Ugandan Government in the 2015/16–2019/20 Health Sector Development Plan, 75% of the population lives within five kilometers of a health facility, with the target of having 85% coverage by the year 2020 [27].

**Table 1:** Ugandan health unit levels, with population capacity of each health unit level and services offered from the Uganda Ministry of Health [26]

Level	Population Capacity	Services Offered
Health Center II	5000	“Preventative, promotive, and outpatient curative services, and emergency maternal deliveries
Health Center III	20,000	All the above services. In addition, provides inpatient, maternal, and laboratory services
Health Center IV	100,000	All the above services. In addition, provides emergency surgery, blood transfusion, laboratory services. Also, supervises levels 2 and 3.
General Hospital	500,000	All the above services, but more comprehensive than HC 4. In addition, provides medicine, surgery, obstetrics, and gynecology, pediatrics, family medicine, and X-ray (plane and mobile)
Regional Referral Hospital	2,000,000	All the above services. In addition, provides specialized services (Medicine, Surgery, Obstetrics, and Gynecology, Pediatrics, ENT, Ophthalmology, Orthopedics, Anesthesia, Pathology, Psychiatry, Dentistry, and Community Medicine. Have specialists, train nurses, have a blood bank, do basic and applied research and provide engineering services to facilities in its health zone.
National Referral Hospital	10,000,000	All the above services, but more comprehensive and advanced than regional hospital. For instance, national hospitals offer advanced diagnostic services such as MRI and CT scans; they have super-specialists, and train doctors, pharmacists, dental surgeons, and graduate nurses and carry out advanced research.”

Uganda has a high level of out-of-pocket health expenditures at 40%, 25 percentage points higher than recommended WHO levels for out-of-pocket expenditures. High out-of-pocket expenditure has led to a high incidence of catastropic health spending among the poor in Uganda. Recognizing the increasing amount of catastrophic spending [28], the Ugandan Government has put forth plans for a National Health Insurance Scheme from taxes paid from individuals in both the formal and informal sector [27], although this scheme has not yet been implemented [29]. Health insurance coverage in Uganda remains low, with private insurance coverage concentrated among individuals living in urban areas, covering approximately 1% of the total Ugandan population [29]. The government provides subsidies to private health providers to lower user-fees, however the subsidies cover just 10 to 20% of operational costs [29]. Approximately 26 community-based health insurance schemes (CBHI) exist in Uganda, the majority of which are in the southern portion of the country [28]. However, just 5 to 10% of the population where the CBHI schemes exist are enrolled. Due to a lack of progressive pooling mechanisms and subsidies for the poor, the poorest Ugandans are generally excluded from coverage since they cannot afford the CBHI premium fees [28]. Care has been provided in government run, public health facilities for free since the abolition of user fees in 2001 [30, 31], but informal payments still may exist [29].

Ambulances are not widely available in Uganda, and emergency medical services generally suffer from a lack of policy coordination, funding, and guidelines [32]. Police vehicles and citizen bystanders are the most common form of emergency transportation, highlighting the resource challenges in providing robust emergency services [32], which is likely rarer in rural areas due to less police presence and infrequent patrol.

### Approach

#### Health-seeking behavior associated with wealth index analysis

The 2016 Uganda Demographic and Health Survey (DHS) [33] was analyzed to identify which health facilities (public or private) the poorest Ugandans mainly utilize. According to Table S1, approximately 88.13% of Ugandans in the poorest quintile and 81.46% of Ugandans in the second poorest quintile receive their health care through government facilities. Table S2 highlights the ownership of transportation means from the DHS survey. Approximately one-quarter of the poorest quintile of Ugandan households own a bicycle, and just 35.46% of the second poorest quintile of Ugandan households own a bicycle. Motorcycle ownership remains extremely low in the poorest quintiles, while car and truck ownership are almost non-existent. As poorer quintiles likely struggle to afford public transport, transportation access to social services is probably low.

#### Geospatial data preparation

The data used in this analysis are listed in Table 2. Spatial data were prepared in ArcGIS Pro [40] and the statistical program, R [41]. All raster data files were up sampled to the largest spatial resolution of all original raster images. Road and health facility data were checked for accuracy and completeness, after which there were noticeable omissions to road layer completeness, especially in northeastern Uganda. To correct omissions within the layer and to verify overall accuracy, all roads in the country were checked and corrected on a parish-by-parish basis using a current, 2020 cloud-free Landsat 8 15 m panchromatic mosaic of Uganda. Road type was defined using the classification outlined in Ouma et al. [42], where primary roads were large, paved highways which connect international borders, secondary roads fed into or were connected to primary roads and connected major towns around the country, and tertiary roads were connected to secondary roads and connected small towns or market centers. A health facility dataset was then obtained from the Uganda Ministry of Health. The dataset was checked for accuracy and completeness using a national health facility inventory and no major issues were found.

**Table 2:** List of variables used in the accessibility analysis

Variable	Data Type	Resolution	Citation
Digital Elevation Model	Raster	90 m	[34]
2020 UN Adjusted Human Population Count	Raster	1 km	[35]
Roads	Vector	N/A	[36]
2016 Sentinel-2 Landcover	Raster	30 m	[37]
Health Facility Locations	Vector	N/A	[38]
Protected Areas	Vector	N/A	[39]

#### Health facility accessibility analysis

Travel time to government health facilities was calculated in the program AccessMod 5 [43] using the Accessibility Analysis function. Protected areas were defined as barriers, as movement and human influence through protected areas is restricted, other than major public roads transecting the park, which were included. A raster surface was created, on a cell-by-cell basis, to calculate travel time to six government health facility types: 1) HC II (Parish-level), 2) HC III (Sub-County-level), 3) HC IV (County-level), 4) General Hospital (GH), 5) Regional Referral Hospital (RRH), and 6) National Referral Hospital (NRH). Higher level facilities were included in lower level facility analyses since the services provided at lower level health services can be provided at higher level health facilities. Three-scenarios were calculated for each of the six health facility types based on walking, bicycling, and driving scenarios, outlined in Table 3. Scenario travel speeds were aligned with those used in Ouma et al. [42], however, the analysis in this paper varied land cover speeds. Speeds of all land covers were varied by − 20 and + 20% to obtain an interval of speed to account for travel time variability and delays [32].

**Table 3:** Travel times in kilometers per hour by landcover for walking, bicycling, and driving scenarios

Landcover	Speed (km/hr)		
	Walking Scenario	Bicycle Scenario	Driving Scenario
Primary Road	6	12	100
Secondary Road	6	12	50
Tertiary Road	6	12	30
Forests	2	2	2
Shrublands	2	2	2
Grassland	5	5	5
Cropland	4	4	4
Regularly Flooded Areas or Aquatic Vegetation	0	0	0
Sparse Vegetation and Mosses	3	3	3
Bare Areas	5	12	5
Built up Areas	5	12	5
Open Water	0	0	0

#### Hotspot cluster analysis

GeoDa v1.8.16.4 [44] was used to calculate the ${\mathrm{G}}_{\mathrm{i}}^{\ast}\left(\mathrm{d}\right)$ statistic [45] to locate spatially significant clusters of low and high health care access by Parish to the nearest government health facility within Uganda using parish boundaries obtained from the Uganda Bureau of Statistics [24]. The analysis was based on three different queens-case contiguity weights matrices on a 1st through 3rd order. A critical distance for each significant cluster (*p*-value <= 0.001) was used. Since the significance of clustering must increase with increasing order [46], the ${\mathrm{G}}_{\mathrm{i}}^{\ast}\left(\mathrm{d}\right)$ statistic (and therefore significance) for each increasing distance must be greater than the ${\mathrm{G}}_{\mathrm{i}}^{\ast}\left(\mathrm{d}\right)$ statistic at all lower orders for each polygon to be considered a significant cluster at higher orders.

After locating significant clusters, descriptive analysis was conducted using a bubble plot of the relationship between time to health facilities and the most recent Multidimensional Poverty Index (MPI) dataset from 2011 [47] to understand if there is a relationships between poverty and significant clusters. Due to the unfortunate temporal data limitation of the MPI data, the author makes explicit the assumption that poverty reduction has occurred roughly uniform among communities in Uganda between 2011 to 2020, although poverty has reduced at a slower rate in northern and eastern Uganda [48]. The MPI is a weighted metric based on three dimensions: 1.) Health (nutrition and child mortality), 2.) Education (years of schooling and school attendance), and 3.) living standards (cooking fuel, sanitation, drinking water, electricity, housing, and assets). The MPI takes into account both the incidence and intensity of deprivation in an area and was used instead of standard, income-based measures of poverty due to its wider definition of deprivation, allowing comparisons between diverse regions of a country (rural vs. urban, ethnic groups, income source, etc.) [49]. Bubble size illustrates total 2020 United Nations adjusted population count data [35] of each parish to analyze relative population compared to health care access and MPI.

#### Statistical modeling data preparation

Five thousand random geographic points were created across Uganda using ArcGis Pro [40] (restricted to the study area used in the health facility accessibility analysis) to extract raster values from raster data layers used in the Random Forest analysis. Pixel values were extracted from a total of 15 data layers, including the MPI dataset used in the hotspot analysis (described above), a 2020 UN-adjusted Human Population Density dataset [50], a rasterized subregion shapefile obtained from the Uganda Bureau of Statistics (11 sub-regions in total) to help account for spatial autocorrelation [24], time in minutes walking to the nearest government health facility for each of the six health unit levels, and time in minutes bicycling to the nearest government health facility for each of the six health unit levels. A “minutes saved by bicycling versus walking” layer was then derived by subtracting the bicycling scenario for each health-unit level from the walking scenario at each health-unit level. The population density and poverty variables were both skewed, with population density exhibiting a strong positive skew and poverty exhibiting a moderate negative skew. The ‘usdm’ package was used for feature selection to check for multicollinearity using the Variance Inflation Factor (VIF). VIF for all model terms for each response variable was below 3, which was below the recommended VIF of 10 or less to rule out multicollinearity [51]. The data were then split into training and test sets based on a 85% training and 15% test split, resulting in 4250 training samples and 750 test samples.

#### Random forest modeling

Conditional Inference Trees [52] were used to model the relationship between population density [50], poverty [47], and health care access in Uganda. Two response variables were used:

1. time in minutes to the nearest government health facility based on the walking scenario (hereafter referred to as the “walking” scenario); and
2. the travel time in minutes reduced to the nearest health facility using a bicycle instead of walking (hereafter referred to as the “time-saved bicycling” scenario),

Sub-regions (Fig. 1) were included in the model to account for spatial-autocorrelation [24]. The ‘cforest function in the Party package [52–54] in the statistical program R [41] was used to generate Conditional Random Forests to separately examine the relationship between the two response variables and the predictor variables of population density, poverty, and sub-region. Twelve Conditional Random Forest models (one model for each health facility level for both the walking scenario and the time-save bicycling scenario) were generated. Model selection was performed by varying the mtry (the number of variables randomly sampled at each split) and ntree (the number of individual trees) cforest hyperparameters to achieve the lowest Mean Absolute Error (MAE; a measure of the average absolute errors between the predicted value and the true value) based on out-of-bag samples [55]. The final-tuned model was then tested and assessed based on a prediction of an independent test set of 750 observations.

Conditional variable importance was used to assess the relative importance of each predictor variable using mean decrease in accuracy, as measured over 500 unbiased conditional inference trees and averaged across twenty importance iterations [54]. Lossless smoothed partial dependence plots were generated using the ‘pdp’ package [56] to illustrate the average impact of poverty and population density on the two response scenarios. Outliers were trimmed from the continuous predictors during partial dependence calculation using a simple boxplot method. Model prediction accuracy was summarized and assessed using the MAE, Root-Mean Square Error (RMSE; the average of the square root of the squared residuals), and the Coefficient of Determination (R^2^; the proportion of variance explained by the predictors regarding the response).

#### Linear mixed-effects modeling

Linear Mixed Effects Models (LMMs) [57–59] were used to compare and validate the performance and interpretations of the Random Forest Models. LMMs were used instead of multiple linear regression to account for geographic data that are spatially-autocorrelated [60]. The training sample of 4250 points was used to fit LMMs on the same predictor and response variables used to fit the Random Forest models. Normality was checked using histograms and normal QQ plots for the population density and poverty predictor distributions. As both variables were highly skewed, Tukey’s Ladder of Powers [61] was used to transform the variables to a normal distribution, after which the transformed variables were centered to increase interpretability. An interaction term between population density and poverty was then calculated from the transformed data. A baseline, null model was first fit to test for significance of the sub-region grouping variable to verify mixed-effects modeling was necessary due to spatial autocorrelation [59]. Due to the significance of the sub-region grouping variable, LMMs were fit for all possible combinations of poverty, population density, and the sub-region cluster variable. The model with the lowest Bayesian Information Criterion (BIC) was selected for each health facility level for both the walking scenario and the time-saved bicycling scenario response variables. The prediction accuracy of the 12 final LMMs was calculated using the independent test set of 750 observations using MAE, RMSE, and R^2^ to allow for direct comparison to the Random Forest model’s accuracy metrics.

## Results

### Health facility accessibility analysis

**Fig. 2:**
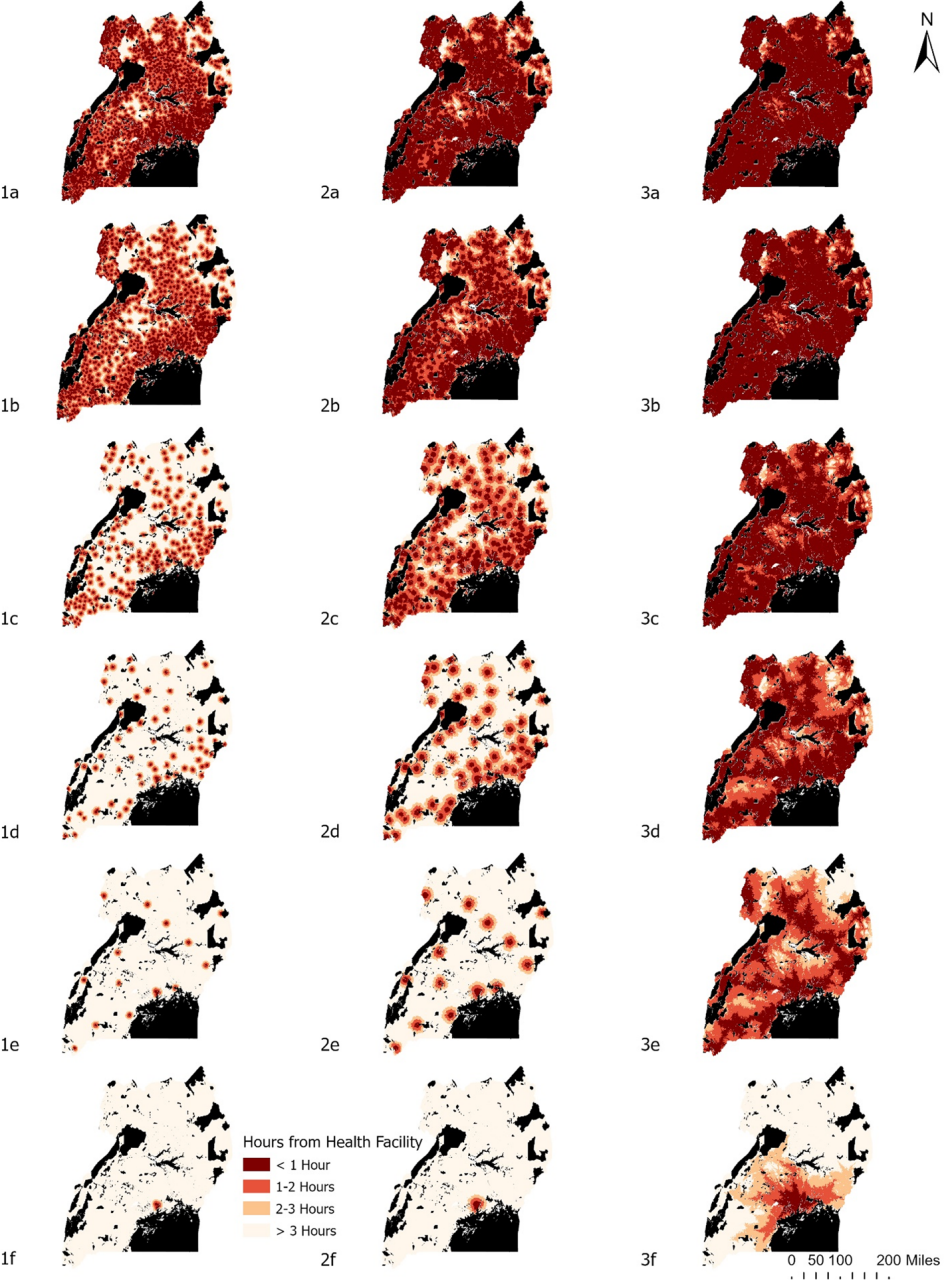
A geographic representation of health care travel times for each health care level: **a** HC II, **b** HC III, **c** HC IV, **d** Hospital, **e** Regional Referral Hospital, **f** National Referral Hospital for the walking (1), bicycling (2), and driving (3) scenarios

The total population proportion within one-hour travel to HCs varied substantially based on the health unit type and travel scenario (Table 4), as well as geographic region (Fig. 2). The proportion of Ugandans within a one-hour walk from their nearest government HC is 71.73%, increasing to 90.57 and 97.10% based on the bicycling and driving scenarios, respectively. The proportion of Ugandans with one-hour access of the nearest HC at each health unit level decreases as one moves from HC II up to NRHs, as well as from faster to slower modes of transportation. This pattern holds throughout all geographic regions of Uganda, although the most limited access appears in the northern and eastern portions of the country. The bicycle scenario appears most beneficial to the access of HC III, with 27.52% of Ugandans increasing their one-hour access to HC III’s. The inclusion of bicycles increased the proportion of Ugandans within one-hour of travel to their nearest government HC from 71.73 to 90.57%.

**Table 4:** Percentage of population located within 1 h for each health unit level for the walking, bicycling, and driving scenarios. -20 and + 20% variable speed results located in parentheses

Health Unit Level	Walk	Bicycle	Drive
Health Center Level II	71.73% (60.01–79.69)	90.57% (83.68–97.96)	97.10% (95.28–97.64)
Health Center Level III	53.05% (47.56–54.55)	80.57% (70.03–87.17)	96.20% (93.36–97.06)
Health Center Level IV	27.85% (22.80–33.00)	46.56% (36.40–56.11)	90.89% (83.68–94.02)
General Hospital	13.69% (11.50–14.84)	24.83% (19.81–30.25)	76.37% (64.42–83.49)
Regional Referral Hospital	7.59% (6.08–9.14)	13.87% (11.97–15.85)	53.42% (41.19–64.42)
National Referral Hospital	5.21% (4.61–5.22)	7.74% (6.98–8.57)	15.90% (14.04–17.64)

### Hotspot cluster analysis

**Fig. 3:**
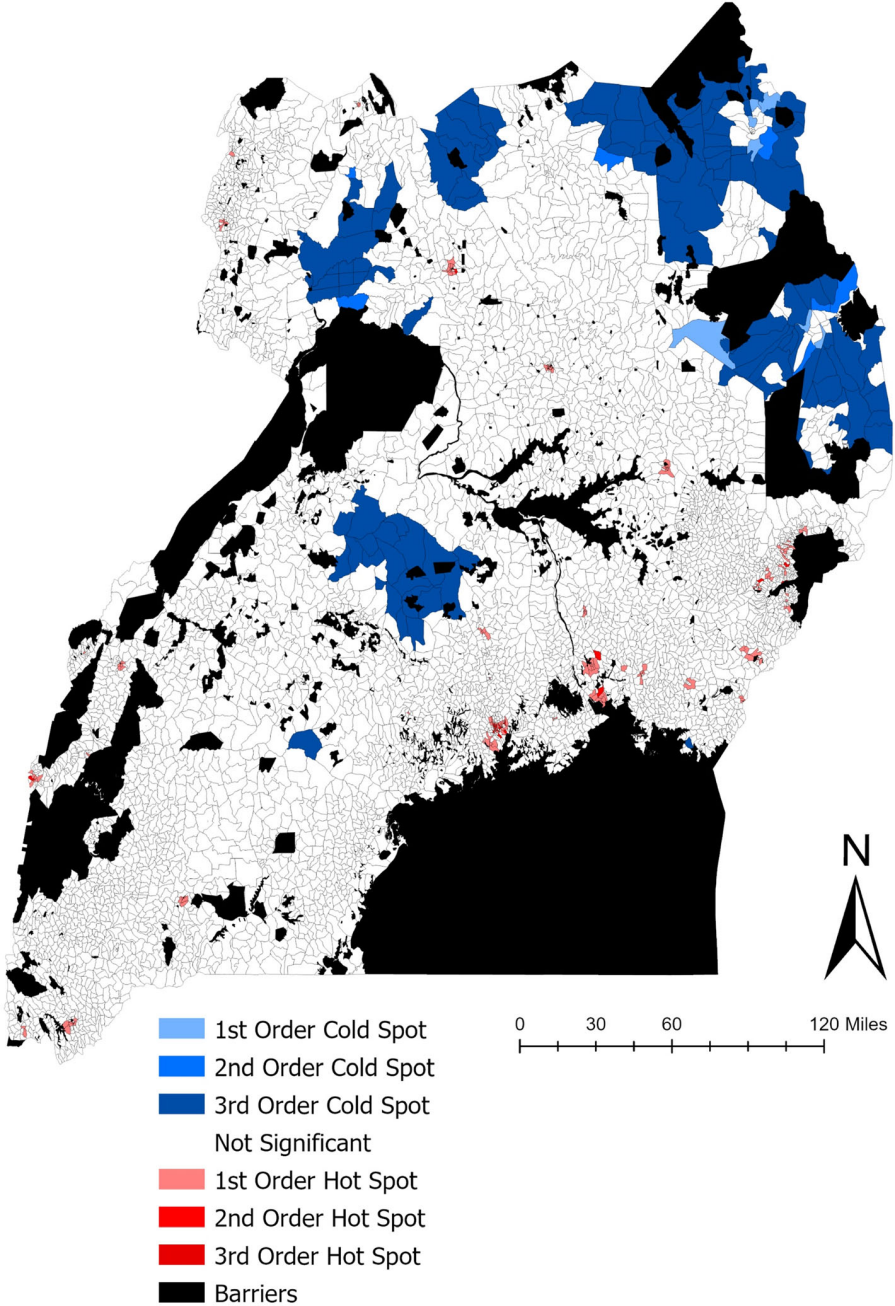
Statistically significant clusters of high (red) and low (blue) health care access within Uganda at a Parish level for the nearest health center

Statistically significant clusters of travel time to the nearest government HC (Fig. 3) confirms the visual assessment of health facility disparities in northern, central, and eastern Uganda. Significant clusters of health care deprivation are of the 3rd order, meaning they are significant at a relatively large distance from the Parish under study. Statistically significant clusters of fast health care access are mainly located surrounding major cities within the country.

Significant clusters of high travel times to health care facilities coincide with higher levels of relative poverty (Fig. S1), exhibited by the blue parish clusters occurring at higher average travel time to health facilities, ranging from 30 min to more than 5 h. When analyzing the impact of bicycles on access to health facilities, travel time to health facilities reduced the most in relatively poorer parishes, including those that were statistically significant clusters (Fig. S2). Low access to health facilities occurs in both high and low populated parishes, represented by the size of the circles in the bubble plot.

### Random forest modeling

**Fig. 4:**
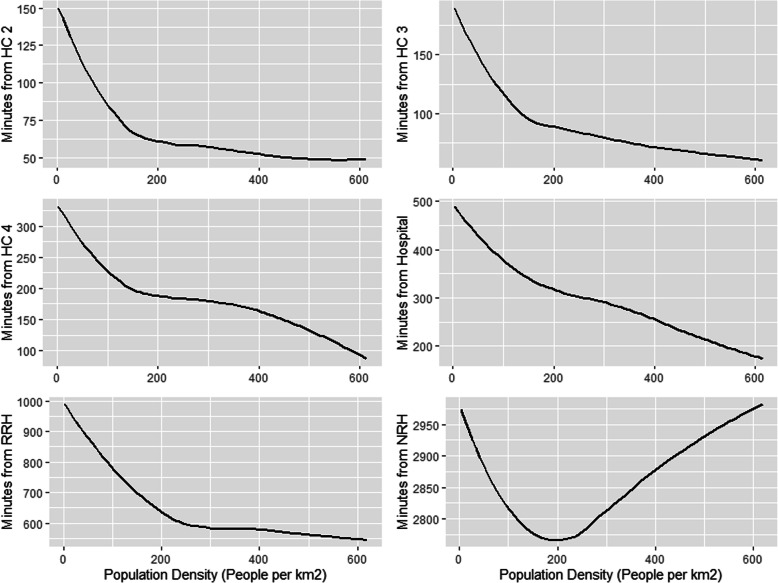
Partial Dependence Plot for travel time in minutes to health clinic by population density for the walking scenario

**Fig. 5:**
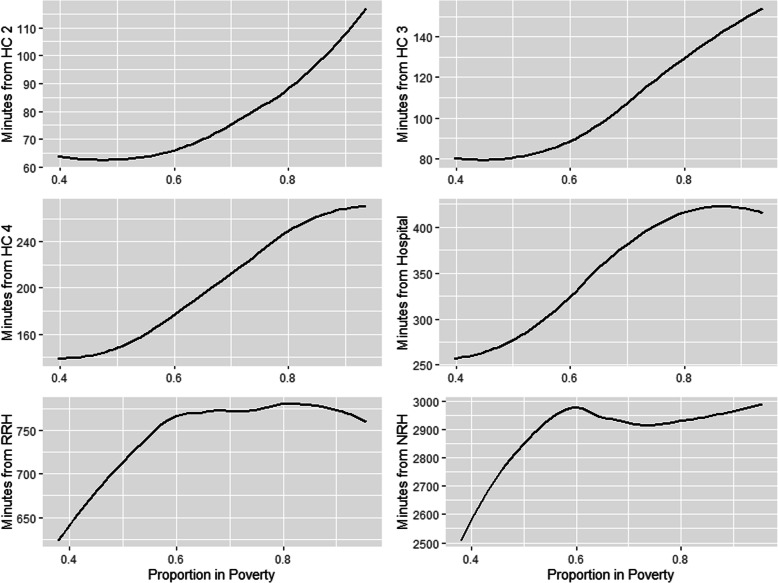
Partial Dependence Plot for travel time in minutes to health clinic by proportion of pixel in poverty for the walking scenario

**Fig. 6:**
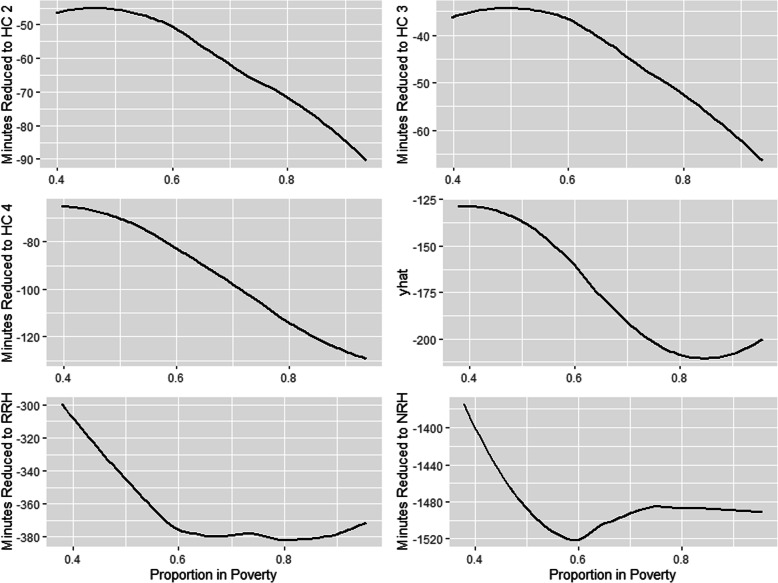
Partial Dependence Plot for minutes reduced by bicycle to health clinic by proportion of pixel in poverty

**Fig. 7:**
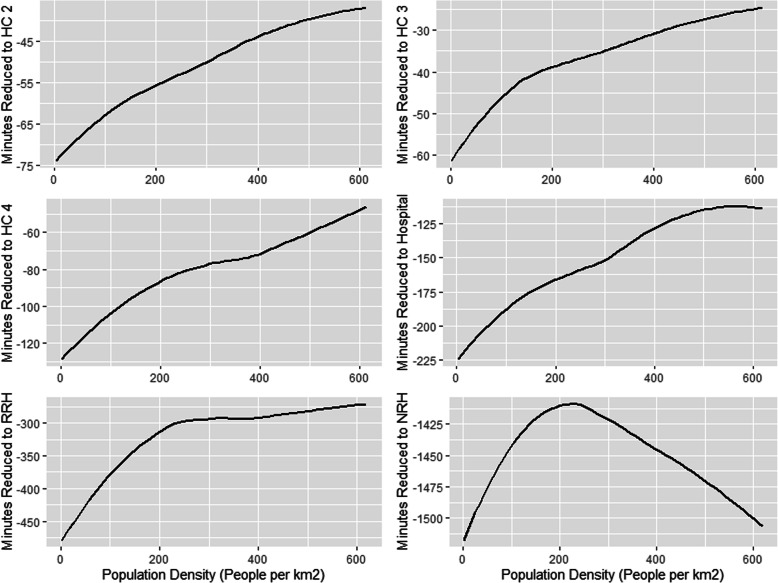
Partial Dependence Plot for minutes reduced by bicycle to health clinic by population density

**Fig. 8:**
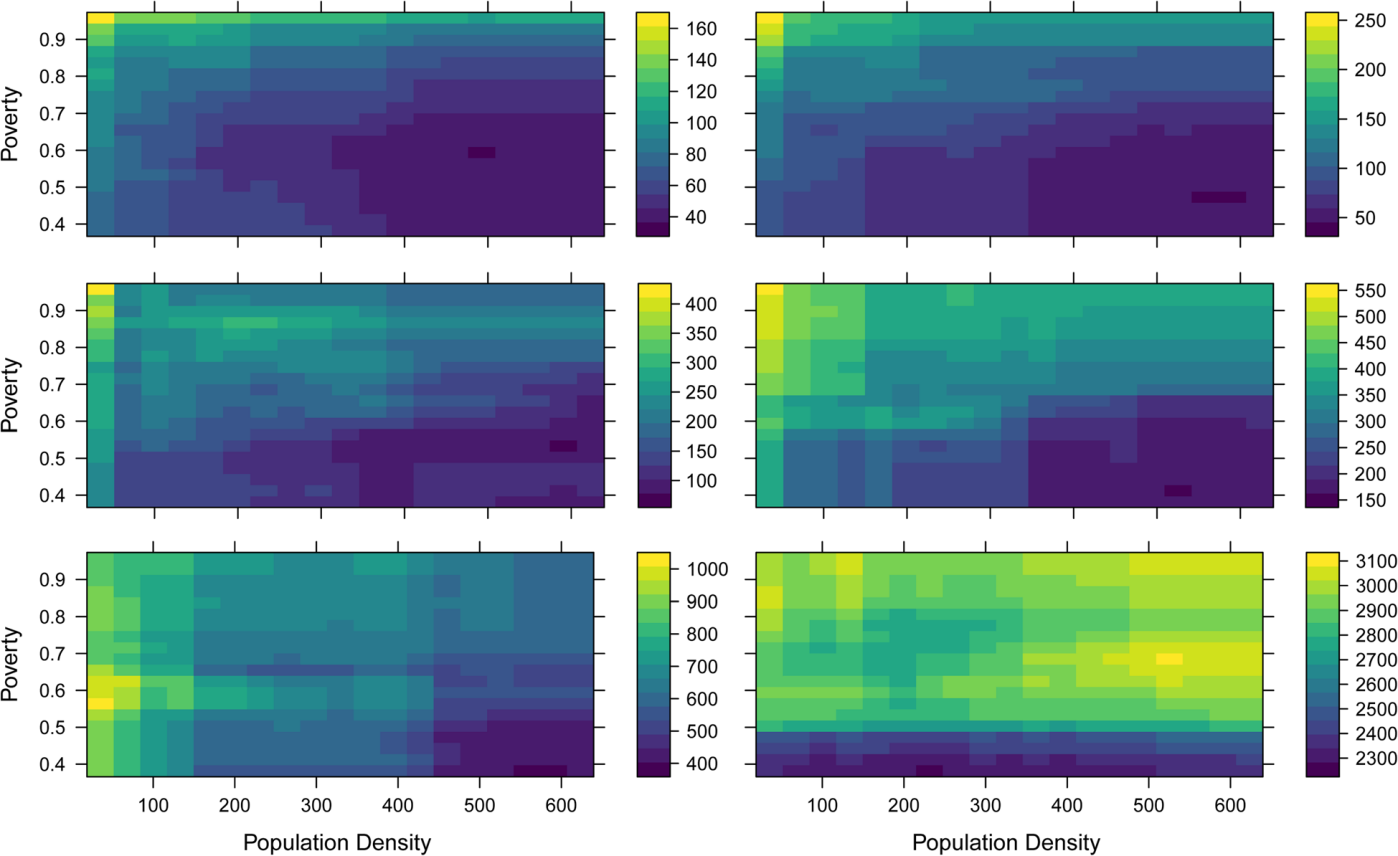
Partial Dependence Plot for travel time in minutes to health clinic for the interaction between population density and poverty for the walking scenario

**Fig. 9:**
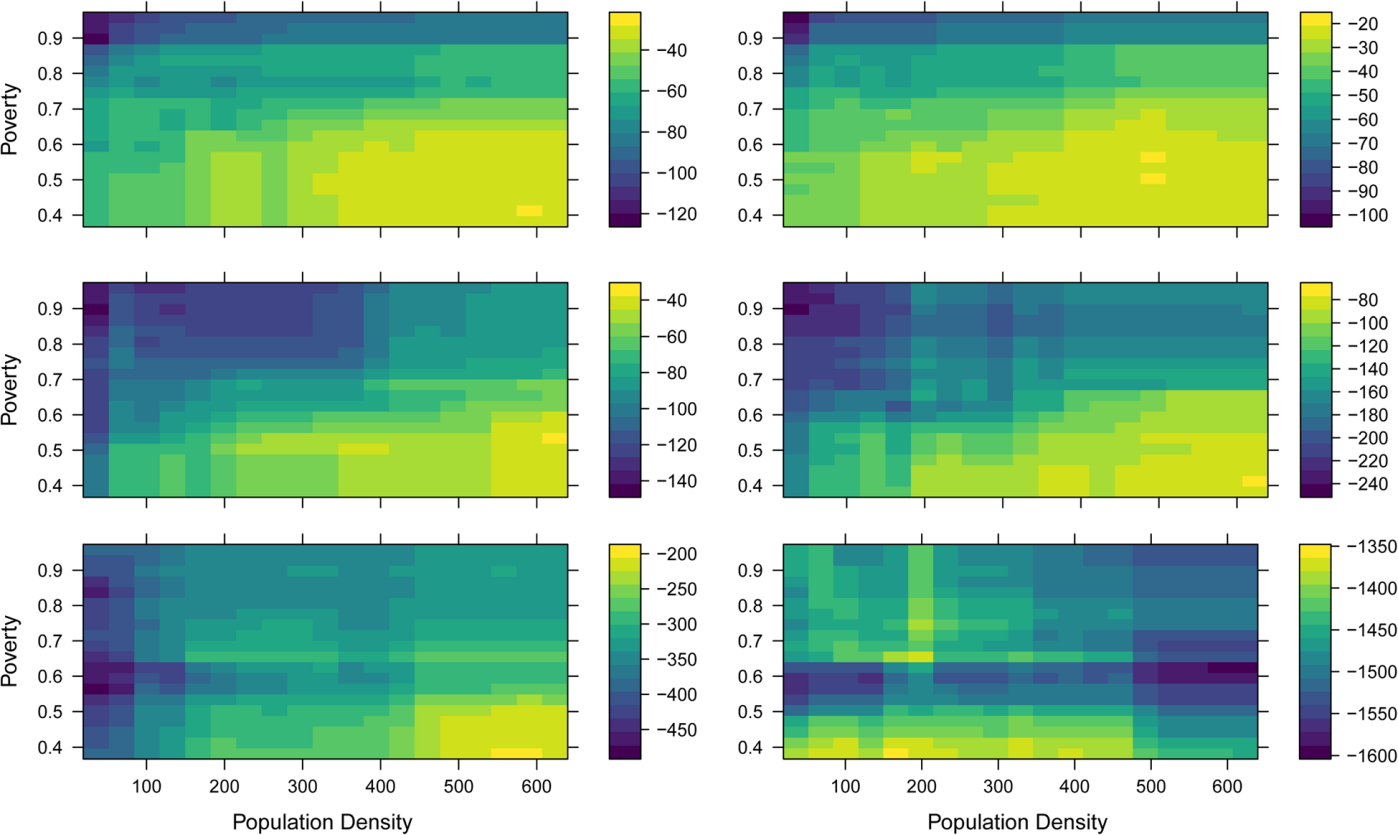
Partial Dependence Plot for minutes reduced by bicycle to health clinic for the interaction between population density and poverty

Population density was the most important variable among all models except for the NRH models, where sub-region was the most important (Table 5). The second most important variable was poverty, except for NRH models where it was population density. For all models except the NRH models, there is an inverse relationship between increasing population density and increasing travel time to health facility (Fig. 4). The NRH model does not follow this general trend, likely due to all NRHs being in Kampala (the capital city), which means both urban and rural areas far from Kampala lack quick walking access to NRH’s. In contrast, there is a direct relationship between increasing travel time to health facilities and the proportion of residents in poverty, including NRHs (Fig. 5). For instance, while areas with approximately half of the population in poverty had an average partial dependence of less than 60 min to a HC II, areas with a proportion in poverty of one had an average partial dependence of more than 110 min. A similar pattern is observed in all other HC levels. Similar patterns are observed between poverty, population density, and the time saved bicycling scenarios. There is a direct relationship between poverty and most average time saved on bicycling to all HC levels (Fig. 6). There is an inverse relationship between population density and time saved bicycling for all HC levels, except NRH’s (Fig. 7). There is a strong inverse relationship on the interaction between poverty and population density on increasing travel time to health facility for all health unit levels except the NRH, based on interaction partial dependence plots (Fig. 8). The interaction partial dependence plot highlights how time to health facilities is highest for those in areas of lower population density with a higher prevalence of poverty. This interaction holds true for the time saved bicycling scenario, where bicycles reduce travel time to health facilities the most in areas with a high proportion of poverty and lower population density across all health facility types, except for NRHs (Fig. 9).

**Table 5:** Mean decrease in accuracy for conditional variable importance values and ranking (in parentheses) for the Population Density, Poverty, and Sub-Region variables in each model. Mean Average Error (MAE), Root Mean Squared Error (RMSE), and the Coefficient of Determination (R^2^) is reported for each model. Scenario 1 represents the time in minutes walking to the nearest health facility, while scenario 2 represents the time in minutes reduced to health facility using a bicycle instead of walking

		Importance Average (rank)			Accuracy Metrics			
**HC Level**	**Scenario**	**Population Density**	**Poverty**	**Sub Region**	**MAE**	**RMSE**	**R**^**2**^	**Mtry**
HC II	1	1018 (1)	457 (2)	96 (3)	30.96	44.47	0.54	2
	2	274 (1)	265 (2)	103 (3)	21.42	30.12	0.41	2
HC III	1	1346 (1)	988 (2)	221 (3)	39.18	53.64	0.54	2
	2	183 (1)	174 (2)	50 (3)	17.6	23.22	0.45	2
HC IV	1	5904 (1)	2248 (2)	250 (3)	67.53	88.95	0.5	3
	2	876 (1)	455 (2)	180 (3)	30.85	39.3	0.49	3
Hospital	1	13,657 (1)	5253 (2)	3208 (3)	105.77	139.37	0.52	3
	2	2729 (1)	1356 (2)	850 (3)	51.43	68.74	0.49	3
RRH	1	68,744 (1)	17,289 (2)	12,533 (3)	158.22	215.48	0.69	3
	2	13,658 (1)	4170 (2)	2733 (3)	84.42	111.87	0.63	2
NRH	1	104,105 (2)	37,625 (3)	377,319 (1)	297.31	426.44	0.9	2
	2	29,039 (2)	10,445 (3)	95,322 (1)	122.68	170.74	0.93	3

### Linear mixed-effects modeling

Sixty LMMs were generated (Table S3). Sub-region was a significant clustering variable, and therefore was included in every model of the analysis as a random effect. The best models (as selected by the lowest BIC) for the walking scenario and the time-saved bicycling scenario are in Table 6 and Table 7, respectively. Generally, all variables, including the interaction between poverty and population density, were significant (*p* < 0.001) for all models, except for the GH walking scenario model and time-saved bicycling HC IV model where the interaction variable was not included in the model, and in the Walking RRH and Bicycling RRH model where both the interaction and poverty variables were not included. According to the best models for the walking scenario (Table 6), increased travel time to all health unit levels is associated with increasing poverty (except for the RRH model, since the variable was not included in the best model). Conversely, high population density is associated with a lower travel time to all health unit levels. The interaction between poverty and population density was also significant (except for the GH and RRH models, since the interaction variable was not included in the best model). Bicycles were associated with the largest reduction in travel time for pixels with a higher proportion of poverty (except for the RRH model, which did not include poverty). Higher population density was associated with a smaller reduction in travel time using bicycles instead of walking for all health unit levels. The interaction between poverty and population density was significant (*p* < 0.001) in all models (except HC IV and RRH, where the interaction was not included).

**Table 6:** Best walking scenario mixed-effects models for each health unit level. Beta coefficient estimate (Estimate), Standard Error (SE), Confidence Interval (CI), t-statistic (t), and *p*-value (p) reported for each fixed effect. Variance (σ^2^), between subject variance (τ_00_), and interclass correlation coefficient (ICC) reported for random effects of each model. Mean Average Error (MAE) and Root Mean Square Error (RMSE) reported as prediction accuracy assessments for each model

	Health Center II					Health Center III				
	*Estimate*	*SE*	*CI*	*t*	*p*	*Estimate*	*SE*	*CI*	*t*	*p*
(Intercept)	74.11	2.50	69.21–79.01	29.66	**< 0.001**	99.44	5.21	89.23–109.66	19.08	**< 0.001**
Poverty	102.20	8.56	85.43–118.98	11.94	**< 0.001**	203.81	10.07	184.07–223.54	20.24	**< 0.001**
Population Density	− 261.91	8.68	− 278.93 –− 244.90	−30.17	**< 0.001**	− 288.61	10.18	− 308.56 –− 268.66	−28.35	**< 0.001**
Interaction	− 628.20	39.88	− 706.36 –− 550.04	−15.75	**< 0.001**	− 725.85	46.34	− 816.67 –− 635.03	−15.66	**< 0.001**
**Random Effects**										
σ^2^	2532.11					3345.50				
τ_00_	59.85 _SubRegion_					286.93 _SubRegion_				
ICC	0.02					0.08				
MAE	33.71					43.97				
RMSE	48.76					58.96				
R2	0.45					0.45				
	**Health Center IV**					**General Hospital**				
	*Estimate*	*SE*	*CI*	*t*	*p*	*Estimate*	*SE*	*CI*	*t*	*p*
(Intercept)	198.92	7.25	184.71–213.13	27.44	**< 0.001**	357.85	17.55	323.46–392.24	20.39	**< 0.001**
Poverty	282.95	16.53	250.56–315.34	17.12	**< 0.001**	485.22	30.37	425.71–544.74	15.98	**< 0.001**
Population Density	− 431.04	16.72	− 463.80 – − 398.28	−25.79	**< 0.001**	− 523.27	30.13	− 582.32 – − 464.22	−17.37	**< 0.001**
Interaction	− 403.72	76.22	− 553.12 –− 254.32	−5.30	**< 0.001**					
**Random Effects**										
σ^2^	9087.88					30,371.98				
τ_00_	545.83 _SubRegion_					3289.19 _SubRegion_				
ICC	0.06					0.1				
MAE	75.86					134.15				
RMSE	97.89					172.46				
R2	0.39					0.26				
	**Regional Referral Hospital**					**National Referral Hospital**				
	*Estimate*	*SE*	*CI*	*t*	*p*	*Estimate*	*SE*	*CI*	*t*	*p*
(Intercept)	726.66	32.19	663.56–789.76	22.57	**< 0.001**	2806.11	326.60	2165.99–3446.23	8.59	**< 0.001**
Poverty						770.02	94.14	585.50–954.53	8.18	**< 0.001**
Population Density	− 1593.59	51.83	− 1695.18 –− 1492.00	−30.74	**< 0.001**	− 821.41	95.07	− 1007.74 –−635.07	−8.64	**< 0.001**
Interaction						1689.33	430.99	844.59–2534.06	3.92	**< 0.001**
**Random Effects**										
σ^2^	100,144.94					286,676.04				
τ_00_	11,077.07 _SubRegion_					1,172,295.43 _SubRegion_				
ICC	0.1					0.8				
MAE	237.56					432.30				
RMSE	310.62					557.29				
R2	0.35					0.83

**Table 7:** Best bicycle-difference scenario mixed-effects models for each health unit level. Beta coefficient estimate (Estimate), Standard Error (SE), Confidence Interval (CI), t-statistic (t), and p-value (p) reported for each fixed effect. Variance (σ^2^), between subject variance (τ_00_), and interclass correlation coefficient (ICC) reported for random effects of each model. Mean Average Error (MAE) and Root Mean Square Error (RMSE) reported as prediction accuracy assessments for each model

	Health Center II					Health Center III				
	*Estimate*	*SE*	*CI*	*t*	*p*	*Estimate*	*SE*	*CI*	*t*	*p*
(Intercept)	−55.42	3.35	−61.99 –−48.86	−16.54	**< 0.001**	−40.55	2.64	−45.72 –−35.38	−15.38	**< 0.001**
Poverty	− 101.74	5.83	− 113.17 –−90.31	−17.44	**< 0.001**	−84.59	4.54	−93.48 –−75.69	−18.64	**< 0.001**
Population Density	95.74	5.89	84.19–107.30	16.24	**< 0.001**	91.61	4.59	82.62–100.60	19.97	**< 0.001**
Interaction	188.73	26.81	136.18–241.29	7.04	**< 0.001**	221.43	20.86	180.54–262.32	10.61	**< 0.001**
**Random Effects**										
σ^2^	1118.22					676.78				
τ_00_	119.50 _SubRegion_					74.02 _SubRegion_				
ICC	0.1					0.1				
MAE	24.88					19.65				
RMSE	34.22					26.21				
R2	0.24					0.30				
	**Health Center IV**					**General Hospital**				
	*Estimate*	*SE*	*CI*	*t*	*p*	*Estimate*	*SE*	*CI*	*t*	*p*
(Intercept)	−91.24	3.84	−98.76 – −83.71	−23.76	**< 0.001**	− 174.03	9.37	− 192.39 –− 155.67	−18.58	**< 0.001**
Poverty	− 123.33	8.06	− 139.12 –−107.54	−15.31	**< 0.001**	− 228.60	15.24	− 258.47 –− 198.73	− 15.00	**< 0.001**
Population Density	151.75	8.00	136.08–167.43	18.97	**< 0.001**	194.28	15.40	164.09–224.47	12.61	**< 0.001**
Interaction						−330.01	70.03	− 467.27 –−192.75	−4.71	**< 0.001**
**Random Effects**										
σ^2^	2153.57					7620.12				
τ_00_	155.30 _SubRegion_					938.09 _SubRegion_				
ICC	0.07					0.11				
MAE	36.64					66.81				
RMSE	46.68					86.18				
R2	0.28					0.21				
	**Regional Referral Hospital**					**National Referral Hospital**				
	*Estimate*	*SE*	*CI*	*t*	*p*	*Estimate*	*SE*	*CI*	*t*	*p*
(Intercept)	− 354.7	15.21	− 384.52 – − 324.89	−23.32	**< 0.001**	− 1427.98	163.75	− 1748.93 –− 1107.04	−8.72	**< 0.001**
Poverty						− 341.30	42.98	−425.53 –− 257.07	−7.94	**< 0.001**
Population Density	706.79	25.92	655.98–757.59	27.27	**< 0.001**	326.25	43.40	241.19–411.31	7.52	**< 0.001**
Interaction						− 1360.50	196.74	− 1746.12 –− 974.89	−6.92	**< 0.001**
**Random Effects**										
σ^2^	25,098.17					59,736.23				
τ_00_	2464.05 _SubRegion_					294,736.58 _SubRegion_				
ICC	0.09					0.83				
MAE	119.80					202.78				
RMSE	155.26					253.47				
R2	0.28					0.85				

### Performance comparison between random forest and linear mixed-effects models

The Random Forest models for each scenario and each health unit level outperformed the LMMs based on the prediction performance on the independent test set of 750 observations, as measured by MAE, RMSE, and R^2^ (Table 8). The Random Forest models highly outperformed the LMMs for the Hospital and the RRH models, with between 0.25–0.35 more variance explained by the RF models compared to the LMMs.

**Table 8:** Difference in Predictive Accuracy of models for Mean Average Error (MAE), Root Mean Squared Error (RMSE), and the Coefficient of Determination (R^2^) between Random Forest Model (Ref) and Linear Mixed-Effects Model for each Health Unit Level and Each Response Scenario. Scenario 1 represents the time in minutes walking to the nearest health facility, while scenario 2 represents the time in minutes reduced to health facility using a bicycle instead of walking

HC Level	Scenario	MAE	RMSE	R^2^
HC II	1	−2.75	−4.29	0.09
	2	−3.46	−4.10	0.17
HC III	1	−4.79	−5.32	0.09
	2	−2.05	−2.99	0.15
HC IV	1	−8.33	−8.94	0.11
	2	−5.79	−7.38	0.21
Hospital	1	−28.71	−33.09	0.25
	2	−15.38	−17.44	0.28
RRH	1	−78.88	−94.62	0.34
	2	−35.38	−43.39	0.35
NRH	1	− 134.99	− 130.85	0.07
	2	−80.1	−82.73	0.08

## Discussion

The results of this paper highlight the time in minutes needed to walk, bicycle, and drive to a HC in Uganda, along with the relationship between poverty, population density, and government health care access. This is the first country-wide analysis in SSA the author is aware of to quantify the difference in access based on walking, bicycling, and driving scenarios for all individual health facility levels. The health facility accessibility analysis showed 71.73% of the Ugandan population to be within a one-hour walk of their nearest government HC. Bicycle ownership was particularly beneficial in increasing the proportion of Ugandans with one-hour access to HC II’s and HC III’s, particularly among poorer areas. Quick access to HCs is significant in urban centers, while many of the rural poor areas across the country experience significant slow access to HC’s. The inequity quantified in this study means that the most vulnerable Ugandans who are least likely to afford transportation experience the highest prohibitive distances to health facilities.

The results of this study align with previous studies on the relationship between geographic access, the rural-urban divide, and socioeconomic status, highlighting inequities in health care access in Uganda [13, 14, 19, 20]. Urban bias was found across all models, with individuals located in more densely populated areas having quicker access to health care, regardless of health facility type. Similarly, health care access time was biased towards areas with higher relative wealth across all government health facility types, with increasing average distance to government health facilities strongly related to higher poverty. The results of this study help support previous quantitative and qualitative work on rural-urban bias in Ugandan government health care [19, 20, 62, 63]. This analysis contributes to the understanding of bicycle ownership and government health care access by demonstrating the disproportionate benefits accrued by poorer communities in Uganda through increasing bicycle ownership and facilitating improved transportation for poorer communities. In general, poorer areas see higher gains in travel time using bicycles compared to relatively wealthy areas. The benefit in bicycle ownership is also demonstrated through the greatest proportional increase in one-hour access to HC III’s compared to the walking scenario.

Increased access to HC III’s is an important finding, as HC III’s are the lowest level HC facility with basic laboratory services, able to provide on-site microscopy and point-of-care testing for HIV and tuberculosis [64]. Even more important, HC III’s are also the lowest level laboratory contact point for the tiered integrated national laboratory system in Uganda [65]. Laboratories are vital to public health efforts for the detection and surveillance of disease and the establishment of biomarkers for chronic disease. However, lack of laboratory access remains an issue in low and low-middle income countries. Through the tiered integrated national laboratory system, samples are collected at HC III’s and transported to a central lab with advanced laboratory diagnostics via a hub and spoke model [65], which increases access to services such as hematology, malaria smears, and CD4 testing for HIV-positive patients at HC III’s.

Faster access to health facilities, as quantified in this analysis, helps improve health equity. The availability of a quicker and/or more affordable means of transport such as a bicycle can empower individuals to seek care at health facilities with higher quality of care, instead of just the health facility that is closest [66]. Helping to ensure greater access to farther facilities can reduce another demand side negative influence on health care in rural Uganda, the perceived poor quality of services [19, 67]. Similarly, the ability to access health care regularly means a patient does not have wait to seek care until their illness is severe, which is especially the case among poorer populations who have a higher burden of disease [63].

Several limitations must be considered while interpreting the results of this study. First, it was not the intention of this paper to advocate for bicycles as the only solution. Bicycles are one tool to increase access and need to be paired with other programs and initiatives that address systemic lack of health care access. Second, this study only investigated geographic access, not affordability or acceptability. While geographic access might be high in specific locations, health care could still be unaffordable or unacceptable for patients, which this study did not investigate. Third, this study did not account for staffing or equipment shortages, which means that health care access for certain regions may have been overestimated where there are equipment stock-outs or low human capital. This is an important point, as staffing shortages remain a significant challenge in Uganda, where there is approximately one doctor and one nurse per every 24,725 and 18,000 people [68], respectively; the majority of whom are concentrated in cities [69]. Further, even though access to ambulances and emergency services are limited and severely underfunded [32], it must be explicitly noted that access to ambulance services was also not included in this analysis. Fourth, seasonality was not accounted for in this study. Due to prolonged wet seasons in Uganda, especially along dirt roads that become saturated and impassible during major rainstorms, travel times could be faster in dry-conditions than wet-conditions. Finally, the delays associated with using public transit were not accounted for within this study. Public transit, including buses and shared taxis, are common forms of transportation over long-distance trips. Both often include long wait-times in taxi and bus parks, as passengers must wait for the bus to fill to capacity before departure. Wait times can be hours long, which would significantly increase the time needed to reach a health clinic using taxis and buses. Similarly, individuals often must walk or take a motorcycle taxi to bus parks or taxi parks prior to departure, which creates an indirect path to the health facilities, through which the study attempted to address through the 20% time-buffer. Another consideration is the data limitation related to spatial Multidimensional Poverty data. As mentioned in the methodology section, the author makes explicit the assumption that poverty reduction has occurred roughly uniform among communities in Uganda between 2011 to 2020, although it must be noted that poverty reduction has occurred at a much slower rate in northern and eastern Uganda than the rest of the country [48]. Coincidently, the statistically significant clusters of low health care access mainly occur in northern and eastern Uganda, which means the relationship between significant clusters and poverty could be stronger than the data illustrates.

## Conclusions

The analysis summarized in this paper found that bicycles decrease travel time for Ugandans to government-run health facilities. Due to disproportionate potential benefits to the poorest Ugandans, bicycles appear to be a pro-poor option, especially for increasing access to HC II’s and HC III’s. Laboratory access is improved via bicycles due to greater access to HC III’s. There is a strong direct relationship between poverty, population density, and time in minutes walking to health facilities, highlighting substantial inequities and an urban bias in government health services in Uganda. This research provides NGOs and social enterprises with evidence of bicycles as a pro-poor intervention for health-care access.

## Supplementary Information

**Additional file 1 Table S1.** The percentage of Ugandans within Demographic Health Survey defined wealth quintiles who use either government or private health centers and/or hospitals, or other/don’t know

**Additional file 2 Table S2.** The percentage of Ugandans within Demographic Health Survey defined wealth quintiles who own a bicycle, motorcycle/scooter, and/or car/truck for the years of 2006, 2011, and 2016.

**Additional file 3.** A summary of all linear mixed effects models generated.

## Data Availability

The datasets used and/or analyzed during the current study are available from the corresponding author on reasonable request.

## Abbreviations

BIC: Bayesian Information Criterion
DHS: Demographic and Health Survey
GH: General Hospital
HC: Health Center
LMM: Linear Mixed Effects Model
MAE: Mean Absolute Error
MPI: Multidimensional Poverty Index
NGO: Non-Governmental Organization
NRH: National Referral Hospital
R^2^: Coefficient of Determination
RMSE: Root Mean Square Error
RRH: Regional Referral Hospital
SSA: Sub-Saharan Africa
VHT: Village Health Team
VIF: Variance Inflation Factor
