# Supplementary material for: Exploring country-wide equitable government health care facility access in Uganda
Source: Int J Equity Health. 2021 Jan 18;20:38. doi: 10.1186/s12939-020-01371-5 (PMC7814723; doi:10.1186/s12939-020-01371-5)
Supplement: Supplementary file 1 — Additional file 1 Table S1. The percentage of Ugandans within Demographic Health Survey defined wealth quintiles who use either government or private health centers and/or hospitals, or other/don’t know [file 12939_2020_1371_MOESM1_ESM.docx]

Table S1: The percentage of Ugandans within DHS defined wealth quintiles who use either Government or Private health centers and/or hospitals, or Other/Don't Know

| **Wealth Quintile** | **Government** | **Private** | **Other or Don't Know** |
| --- | --- | --- | --- |
| Lowest | 88.13% | 11.66% | 0.21% |
| Second | 81.46% | 18.39% | 0.15% |
| Middle | 78.88% | 20.97% | 0.14% |
| Fourth | 69.45% | 30.30% | 0.25% |
| Highest | 41.74% | 58.01% | 0.25% |
