# Supplementary material for: Exploring country-wide equitable government health care facility access in Uganda
Source: Int J Equity Health. 2021 Jan 18;20:38. doi: 10.1186/s12939-020-01371-5 (PMC7814723; doi:10.1186/s12939-020-01371-5)
Supplement: Supplementary file 2 — Additional file 2 Table S2. The percentage of Ugandans within Demographic Health Survey defined wealth quintiles who own a bicycle, motorcycle/scooter, and/or car/truck for the years of 2006, 2011, and 2016. [file 12939_2020_1371_MOESM2_ESM.docx]

Table S2: The percentage of Ugandans within DHS defined wealth quintiles who own a bicycle, motorcycle/scooter, and/or car/truck for the years of 2006, 2011, and 2016.

|  | **Bicycle** | | | **Motorcycle/Scooter** | | | **Car/Truck** | | |
| --- | --- | --- | --- | --- | --- | --- | --- | --- | --- |
| Wealth Quintile | *2006* | *2011* | *2016* | *2006* | *2011* | *2016* | *2006* | *2011* | *2016* |
| Lowest | 24.39% | 26.35% | 27.67% | 0.00% | 0.25% | 1.26% | 0.00% | 0.00% | 0.00% |
| Second | 40.82% | 40.68% | 35.46% | 0.01% | 1.50% | 4.13% | 0.03% | 0.01% | 0.06% |
| Middle | 39.94% | 45.03% | 40.65% | 0.85% | 4.53% | 8.75% | 0.00% | 0.09% | 0.35% |
| Fourth | 48.69% | 49.45% | 40.15% | 2.87% | 12.04% | 17.82% | 0.17% | 0.59% | 1.28% |
| Highest | 34.97% | 26.67% | 22.63% | 9.09% | 18.65% | 20.74% | 7.87% | 12.68% | 15.79% |
